# Toward a Glossary of Self-related Terms

**DOI:** 10.3389/fpsyg.2017.00280

**Published:** 2017-02-28

**Authors:** Alain Morin

**Affiliations:** Department of Psychology, Mount Royal UniversityCalgary, AB, Canada

**Keywords:** self-reflection, mindfulness, mental time travel, self-esteem, self-knowledge, self-concept, Theory-of-Mind, self-regulation

## Abstract

Some scholars have noted that an impressive number of self-related terms have been gradually introduced in the scientific literature. Several of these terms are either ill-defined or synonymous, creating confusion, and redundancy. In an effort to minimize this problem, I present a novel and systematic way of looking at possible relations between several key self-terms. I also propose a tentative classification scheme of self-terms as follows: (1) basic terms related to the overall process of self-perception (e.g., self-awareness), (2) non self-terms that are importantly associated to some other self-terms (e.g., consciousness and Theory of Mind), (3) processes related to the executive self and involving agency, volition, and self-control (e.g., self-regulation), and (4) self-views, that is, the content and feelings about the self (e.g., self-esteem). Three additional categories not discussed in this paper are self-biases, reactions to the self, and interpersonal style. Arguably unambiguous definitions for some of the most important and frequently used self-terms are suggested. These are presented in tables meant for the reader to search for definitions as well as related terms.

## Introduction

Mark Leary offered the following observation in his Editorial for a special issue on “What Is the Self?” in *Self and Identity* (2004, pp. 2–3):

“... perhaps the field will move slowly to embrace a set of precise, clear, and distinct terms for each of the phenomena that we study under the self and identity umbrella... Whatever terms one uses, providing clear and precise definitions will also help to promote communication and minimize confusion.”

Indeed, definition confusion abounds in the field, as exemplified by the interchangeable use of terms such as “extended self”, “meta-representational/conceptual self-consciousness”, and “reflective/recursive/meta-consciousness” to designate self-awareness (Morin, 2006). Although Leary himself and others (e.g., Trapnell and Campbell, 1999; Leary and Tangney, 2003) have attempted to define and classify multiple self-related terms, close inspection of these efforts reveals that (a) they are only partial, that is, they leave out many self-terms undefined, (2) their proposed taxonomy systems are arguably limited and could be expanded, and (3) they neglect to discuss how various self-terms are conceptually and empirically connected. The present paper addresses these limitations by putting forward a broader and more systematic glossary of self-related terms which includes a consideration of how some key self-terms relate to one another. Its main goal is to organize many self-terms (and related ones) into a comprehensive and logical system in order to make more sense of what currently looks remarkably chaotic. Like any taxonomy system it is not perfect (e.g., possibly incomplete) but hopefully acts as a preliminary effort in the right direction.

Leary (2004) establishes a distinction between three types of self-related processes: (a) processes involved in reflexivity and self-awareness, (b) the knowledge, beliefs, and feelings people have about themselves, and (c) processes involved in agency and self-regulation. While this initial classification system is certainly helpful, I suggest that it can be improved as follows (see Morin and Clements, 2015): (1) basic terms related to the overall process of self-perception (e.g., self-awareness, self-reflection), (2) non self-terms that are importantly related to various key self-terms, such as consciousness and Theory-of-Mind, (3) self-processes pertaining to the executive self and involving agency, volition, and self-control, like self-talk and self-regulation, (4) self-views, that is, the content and feelings about the self (e.g., self-esteem, self-efficacy), (5) self-biases used mainly to protect the self, such as self-enhancement and self-deception, (6) reactions to the self, such as self-compassion and self-blame, and (7) interpersonal style, such as self-confident and self-effacing. The present paper exclusively focuses on the four first categories for length purposes; a second, independent, article will discuss the remaining three categories.

Obviously, some self-related terms fit into more than one category: to illustrate, self-knowledge can be understood both as a basic self-perception unit (category 1) and as a self-view (category 4). Also, note that the above taxonomy does incorporate Leary’s original classification system: his self-reflection processes (a) are included in the “basic terms” category (1); his self-knowledge, beliefs, and feelings contents (b) mostly refers to the “self-views” group (3), and his self-regulatory processes (c) are captured by the “executive self” category (2). Note that the purpose of category 2 is to discuss terms that are literally *non*-self terms. All other tables/categories present “genuine” self-terms such as self-knowledge, self-esteem, self-recognition, etc.

## Basic Terms Related To Self-Perception

**Table 1** presents definitions (and in some cases conceptually related terms) of multiple basic terms associated with the overall process of self-perception. To summarize, “self”, which contains all imaginable physical and psychological (e.g., cognitive, affective, motivational, social) characteristics that make a person unique and different from others, represents the most important (and frequently used) umbrella term related to all self-terms to be discussed here. “Self-perception” refers to the overall process of getting access to “self-variables”, including specific self-characteristics such as being tall, habitual behaviors such as being on time for work, and more global and stable conclusions about the self (“self-schemas”), such as being punctual or lazy. “Self-awareness” can be construed as being the most fundamental self-perceptual process of directing attention inward toward the self (Duval and Wicklund, 1972) and actively identifying, processing, and storing information about the self (Morin, in press). It is important to note that an organism can possess a self without knowing about it. That is, one can be effectively processing information about the external environment (“consciousness”; see next section) in an entirely unique and personal way (“self”) without being self-aware.

**Table 1:** Basic terms pertaining to general self-perception.

Term	Related terms	Definition
Self	• Person • Personality • Identity • Character	What distinguishes oneself from others; all imaginable private and public aspects making up who a person is (Morin, 2006)—e.g., thoughts, emotions, goals, values, sensations, memories, traits, attitudes, physical attributes, appearance, behaviors, skills.

Self-perception	See all related terms within **Table 1**	Overall process of self-awareness, self-knowledge acquisition and self-concept formation; an awareness of the characteristics that constitute one’s self (Self-perception, 2016).

Self-variable	• Self-information • Self-representation • Self-schema • Self-knowledge • Self-characteristic	Any process, information or experience related to the self (Ritsner and Susser, 2004); any aspects of the self that can be apprehended through self-awareness (Morin, 2011).

Self-schema		Specific (organized) unit of self-information; small conclusion about the self; cognitive generalization about the self (Markus, 1977).

Self-relevance	Self-referential	Any information that refers to, is relevant to, the self (Shih et al., 2002).

Self-awareness	• Self-directed attention • Self-focus • Self-examination • Self-observation • Introspection • Meta-awareness • Self-consciousness • Self-reflection • Mindfulness	Capacity to become the object of one’s own attention; to focus one’s attention inward toward the self (Duval and Wicklund, 1972; Silvia and Duval, 2001); to actively identify, process, and store information about the self (Morin, 2011).

Self-consciousness	See self-awareness synonyms above	Consistent tendency (trait) to direct attention inward more or less often; also refers to self-awareness with a social evaluation component (Fenigstein et al., 1975).

Self-analysis	• Psychotherapy • Psychoanalysis	Systematic self-examination in a psychotherapeutic context, with a psychoanalytic connotation (Self-analysis, 2016).

Self-reflection	• Self-awareness • Self-consciousness • Self-observation • Mindfulness	Active cognitive act of examining the self; self-focus motivated by curiosity or epistemic interest in the self; intellectual self-attentiveness; healthy form of self-focus (Trapnell and Campbell, 1999; Takano and Tanno, 2009).

Self-rumination	Self-absorption	Negative, chronic, and persistent self-focus motivated by perceived threats, losses, or injustices to the self; neurotic self-attentiveness (see previous term for references).

Self-recognition	• Self-directed behavior • Mirror self-recognition • Mark/rouge test	Self-directed behaviors emitted in front of a mirror; correctly identifying the image of self as self (Gallup, 1968).

Self-knowledge		Organized set of accurate self-information; realistic self-concept; accurate introspection about one’s own self (Gibbons, 1983; Carlson, 2013; Wilson, 2009).

Typically, self-awareness is seen as a transient and situational state induced by environmental experiences (e.g., self-focusing stimuli like mirrors and cameras; Buss, 1980; Davies, 2005). In contrast, “self-consciousness” designates a more permanent personality-like trait supposedly unaffected by external circumstances. It represents a more or less frequent tendency to focus attention on the self (Fenigstein et al., 1975; Scheier and Carver, 1985). Self-awareness can further be divided into two components: “self-reflection” and “self-rumination” (Trapnell and Campbell, 1999). Self-reflection represents a healthy, non-evaluative form of self-attentiveness, where the person enjoys learning new things about the self in order to increase self-understanding and improve the self (“self-regulation”). This greatly resembles “mindfulness”, defined as a non-evaluative, non-elaborative attention to one’s current experience (Carlson, 2013), which will be discussed below. On the other hand, the self-ruminative person gets trapped in a circular, repetitive, negative, and judgmental cycle of anxiety-ridden self-focus that creates a state of self-absorption (Mor and Winquist, 2002). In that state the person becomes excessively self-centered, which negatively impacts her interest in, and compassion for, others [impeded “Theory-of-Mind” (ToM); Joireman et al., 2002; Joireman, 2004].

One particularly contentious issue is the meaning of self-recognition: while some argue that recognizing oneself in a mirror (or on a computer screen) signifies that one is fully self-aware and can engage in ToM (Gallup, 1985; Keenan et al., 2003), others claim that self-recognition simply rests on bodily self-awareness and does not imply access to one’s or others’ mental states (Morin, 2010; Brandl, 2016; Saidel, 2016).

## Related Non-Self-Terms

**Table 2** presents definitions of some non-self-terms that are nonetheless importantly related to various key self-terms. Expanding on a point discussed above, one can be efficiently processing information about the external environment (“consciousness”) without knowing about it (“self-awareness”). Numerous everyday complex actions are properly accomplished without the agent being aware of performing them (see Farthing, 1992), as when one drives back home after work while thinking about something unrelated to driving *per se*. Similarly, one can postulate that most non-human animals are conscious but not self-aware (Morin, 2006). However, all self-related processes require consciousness to be performed. That is, an unconscious organism cannot possibly engage in self-reflection, prospection, ToM, etc., although it has been argued that some forms of mental activity are preserved in the absence of consciousness (Owen et al., 2006).

**Table 2:** Non-self-terms associated with various key self-related terms.

Term	Related terms	Definition
Consciousness	• Awareness • Wakefulness	To focus attention outward toward the environment; opposite of unconsciousness (Natsoulas, 1996).

Metacognition	• Self-awareness • Meta-awareness • Decentering • Cognitive distancing • Mindfulness • Introspection	Being aware of one’s own thought processes (Bernstein et al., 2015).

Introspection	• Self-reflection • Self-awareness • Metacognition	The examination of one’s own conscious thoughts and feelings (Schultz and Schultz, 2012).

Mindfulness	• Self-reflection • Mindedness	Non-evaluative, non-elaborative attention to one’s current experience (Carlson, 2013).

Autobiography	• Mental time travel • Autonoetic consciousness • Episodic memory	Recollection of one’s own past experiences (Conway, 2005).

Prospection	• Mental time travel • Autonoetic consciousness • Future oriented thinking • Episodic future thought • Simulation • Projection	Ability to simulate personal future episodes (Szpunar et al., 2016).

Death awareness		Knowledge that one will unavoidably die; presupposes self-awareness (Gallup, 1998).

Theory-of-Mind	• Inferring mental states • Mentalizing • Intentional stance	Thinking about others’ mental states (e.g., needs, thoughts, desires, emotions) (Frith and Frith, 2003).

Although “metacognition” involves self-attention and is closely connected to other terms such as “self-awareness” and “introspection”, one must keep in mind that it refers to attention exclusively paid to one’s thoughts—private, unobservable self-aspects. Metacognition thus excludes attention focused on any other type of private self-aspects like emotions, goals, sensations, memories, as well as public self-aspects such as appearance, tone of voice, others’ opinion of oneself, etc. It is tempting to use “mindfulness” and “self-reflection” interchangeably, but Carlson (2013) sees subtle differences between these two constructs (E. Carlson, personal communication, January 20, 2014). Both mindfulness and self-reflection are considered healthy forms of self-focus based on genuine curiosity about the self, but mindfulness also involves “decentering” or distant observation (a third-person perspective on the self), accepting what is observed in a compassionate way, and non-attachment/reactivity. Regarding ToM development, the most accepted proposal is the Simulation view according to which one uses one’s knowledge of one’s internal states in order to imagine (“simulate”) what it could be like for others to experience comparable states (Gallup, 1985; Hesslow, 2002; Dimaggio et al., 2008; Focquaert et al., 2008). This view suggests that self-awareness comes first and that a disruption in self-awareness will negatively affect ToM—a prediction at least partially supported by neuropsychological observations (see Bivona et al., 2014). Although “autobiography” and “prospection” refer to mental time travel in opposite temporal directions—past and future respectively—, a large body of research suggests that they are intimately linked. Deficits in autobiographical access following brain injury or dementia reliably result in prospection deficits; common brain activations are observed when participants either engage in autobiographical or prospection tasks (for a review see Szpunar et al., 2016). This evidence supports the notion that the primary function of episodic memory is to provide building blocks from which episodic future thoughts are constructed. The contents of episodic memory may be sampled and recombined in various ways in the course of constructing a coherent mental representation of novel future scenarios (Szpunar, 2010).

## Self-Processes

**Table 3** presents definitions (and related terms whenever possible) of processes used by the self as an executive agent. Executive functions represent a set of cognitive processes that are involved in the control of behavior—selecting and successfully monitoring behaviors that facilitate the attainment of chosen goals. These functions include attentional control, inhibitory control, working memory, and cognitive flexibility, as well as reasoning, problem solving, and planning (Diamond, 2013).

**Table 3:** Self-processes used by the self as an executive agent.

Term	Related terms	Definition
Self-regulation	• Self-control • Self-improvement • Free will • Volition • Agency	Altering one’s behavior, resisting temptation, changing one’s mood, selecting a response from various options, filtering irrelevant information (Baumeister and Vohs, 2007).

Self-agency	• Self-determination • Autonomy	Subjective awareness that one is initiating, executing, and controlling one’s own volitional actions in the world (Jeannerod, 2003).

Self-discipline	Self-control	The actual act of doing things that increase the likelihood of successful self-regulation (Self-discipline, 2016).

Self-talk	• Inner speech • Private speech • Self-directed speech • Subvocal/covert/acommunicative speech • Auditory imagery • Speech-for-self • Propositional thought • Self-verbalizations • Internal dialog/monolog • Sub-vocalizations • Self-statements • Silent verbal thinking	Talking to oneself either silently or aloud (Morin, 2012).

Self-description		Non-evaluative list of self-aspects (Higgins, 1996).

Self-evaluation	Self-assessment	Comparison between real and ideal selves (Higgins, 1987; Sedikides, 1993).

Self-presentation	• Self-monitoring • Self-management • Impression formation	Manipulating the impression one makes on others (Martin and Leary, 1999).

Self-monitoring	• Self-affirmation • Self-promotion	Actual active process of self-management; careful self-focus aimed at making a good impression (Snyder and Gangestad, 1986).

Self-disclosure	Openness	Willingness to open up to others and share self-information (Cozby, 1973).

Self-actualization	Actualization	Realization or fulfillment of one’s talents and potentialities (Maslow, 1962).

It is safe to posit that most self-related processes heavily rely on “self-talk” (Morin, 2005, in press; DeSouza et al., 2008; Neuman and Nave, 2010; Turjman, 2016). That is, processes such as self-reflection, self-rumination, self-evaluation, self-regulation, and self-monitoring require an active verbal conversation with oneself, e.g., “Why did I do that?”, “I should eat less carbs”, “I look good in this outfit”. Indeed, several studies report positive significant correlations between different measures of inner speech and self-related constructs (e.g., Morin et al., 1993; Brinthaupt et al., 2009). Inner speech loss leads to self-reflection impairment (Morin, 2009). And self-reflective tasks such as deciding if personality traits describe oneself or not significantly recruit the left inferior frontal gyrus, a brain area associated with inner speech production (Morin and Hamper, 2012). Similarly, self-regulation requires self-awareness and self-evaluation (Bandura, 1991): one needs to know what to change about the self (“self-awareness”) in order to effectively modify it (“self-regulation”).

There is no definitive consensus on this issue, but it is common to view “self-regulation” as representing a complex long-term process involving the attainment of numerous goals and sub-goals, such as successfully graduating from university. In contrast, “self-control” refers to a single short-term effort at resisting temptation or delaying gratification, like studying instead of watching a movie. As such, self-regulation is made up of numerous instances of self-control. The person being successful at both self-regulation and self-control is said to possess “self-discipline”.

## Self-Views

**Table 4** depicts different types of content that the self is made up of—thoughts, beliefs, emotions, and evaluations about the self, produced by self-processes discussed in the previous section. At the risk of oversimplifying, the self-concept represents a global, emotionally flat, view that one has of oneself (Who am I?), whereas self-esteem constitutes the emotional evaluation of one’s self-concept (Do I like myself?). Note that self-esteem can also be understood as a reaction to the self (sixth category) in addition to a self-view (fourth category). Furthermore, it is essential to recognize that self-concept and self-knowledge are not interchangeable terms. The self-concept is a potentially, but not necessarily, accurate image of the self; people often hold unrealistic views of themselves as evidenced by the discrepancy between self-ratings on personality inventories and ratings of others who are close to the self (see Carlson, 2013). Self-knowledge can be conceived of as accurate information about the self—a realistic self-concept. As a level of confidence about one’s future performance, self-efficacy evidently shapes one’s self-esteem. Self-esteem represents an important predictor of life outcomes, with a positive self-view being associated with better health, occupational, financial, and relational consequences, and vice-versa (Kuster et al., 2013). Self-esteem increases from adolescence to middle adulthood, peaks at about age 50 to 60 years, and then decreases at an accelerating pace into old age (Orth and Robins, 2014).

**Table 4:** Self-views (content and feelings about the self).

Term	Related terms	Definition
Self-concept	• Self-image • Self-definition	Global (although also situation-specific) perception/image of self; coherent, and organized set of self-information acquired through self-awareness and social feedback/comparison; who one thinks one is (L’Ecuyer, 1978; Marsh and Shavelson, 1985; Hornsey, 2008; Racy, 2015).

Self-esteem	• Self-worth • Self-feeling • Self-respect • Self-liking • Self-judgement	Global emotional evaluation of the self; positive or negative attitude toward the self (Rosenberg, 1965; Greenwald et al., 2002).

Self-efficacy	• Self-confidence • Self-assurance • Self-belief	Level of confidence of being able (or not) to perform a specific task successfully (Bandura, 1977; Alden, 1986).

Possible self	• Ideal self • Ought self • Future self	Self-concept in the future; one’s idea of what one might become, would like to become, is afraid of becoming (Markus and Nurius, 1986).

Self-discrepancy	Self-incongruence	Incongruence between real self and ideal/ought self (Rogers, 1961; Higgins, 1996).

Self-construal		Different understanding of the self (e.g., dependent, independent) in different cultures (Markus and Kitayama, 1991).

Self-categorization		Self-perception as a group member possessing the same characteristics and reactions as other group members (Abrams et al., 1990).

Self-conscious emotions		Emotions that can only be experienced by self-aware creatures—e.g., guilt, shame, embarrassment (Lewis and Ramsay, 2004).

## Relations Between Self-Terms

**FIGURE 1:**
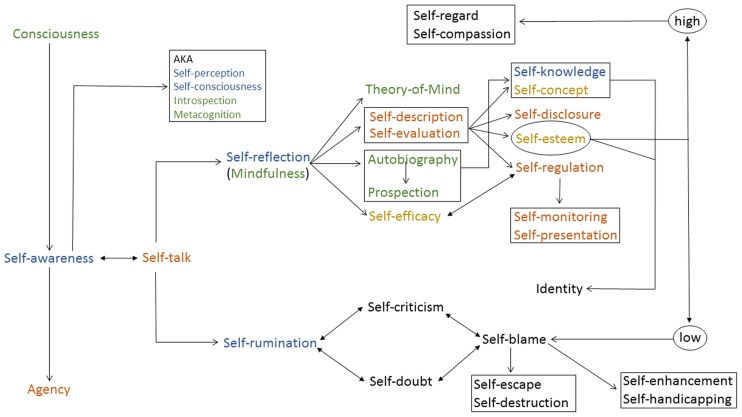
**Postulated relations between various self-related processes.** Colors refer to the following categories: Blue = basic terms; Green = non-self-terms; Red = self-processes; Yellow = self-views.

**Figure 1** shows numerous possible links between several key terms defined in this paper. Its purpose is to go beyond mere definition and classification by exploring how self-terms conceptually may relate to one another. By “conceptually related”, I mean how several key self-processes create additional self-processes which themselves also lead to further self-processes, and so on. This represents a preliminary effort and will no doubt gain complexity and empirical support in the future.

**Figure 1** reads as follows. A conscious organism is awake and interacts with its environment in complex ways for survival purposes; attention is directed outward. When the organism directs attention inward it becomes the object of its own attention—it becomes self-aware. This state creates a sense of agency, an awareness that one is responsible for one’s actions in the world. Self-awareness, in essence a form of communication with oneself, is associated with a verbal and usually silent conversation with oneself—self-talk. This inner voice may help sustain self-reflection or self-rumination, or a combination of both. Self-rumination represents a repetitive, uncontrollable, and negative focus on the self which may lead to self-criticism, self-doubt, and self-blame, and these in turn may feed back into self-rumination and further aggravate the anxious self-thinking, hence the bidirectional arrows connecting these last self-terms. The individual may then resort to self-escape (e.g., watching television; Moskalenko and Heine, 2003) or ultimately to self-destruction (e.g., addiction or suicide; Baumeister, 1991). Alternatively, or simultaneously the person may engage in self-enhancement and/or self-handicapping in order to make oneself feel better about oneself (Strube and Roemmele, 1985). This entire lower branch in **Figure 1** is most probably associated with low self-esteem, as shown on the extreme right portion.

Inner speech may also help sustain self-reflection, where the person focuses on learning about and improving oneself. This non-judgmental form of self-attention arguably represents a prerequisite for the emergence of several key self-related functions: ToM, self-description, self-evaluation, mental time travel, and self-efficacy. Note that the unidirectional arrow connecting autobiography to prospection reminds us that the former serves as a springboard for the latter. The bidirectional arrow linking self-efficacy and self-esteem suggests that one’s confidence (or lack thereof) at performing well on various tasks affects one’s evaluation of the self, and vice-versa. Self-description, self-evaluation, and mental time travel all participate in the gradual construction of a self-concept, the acquisition of self-knowledge, the emergence of self-esteem, self-disclosure, and self-regulation. Self-regulation involves self-monitoring as well as self-presentation. Self-knowledge, self-concept, and self-esteem form the basis of one’s sense of identity. This entire higher branch in **Figure 1** is most probably associated with high self-esteem, as shown on the extreme right portion, and may lead to self-regard and self-compassion.

## Conclusion

An efficient scientific investigation of the self relies on the use of clearly defined terms that describe its multiple underlying operations and functions. One can unfortunately find many examples in the literature of single self-terms being differently defined; another problem consists in the use of different terms to designate the same construct. A unified taxonomy of self-related terms is currently lacking and the present paper offers a preliminary framework in that direction. I propose that a parsimonious yet inclusive classification system should include basic terms related to the overall process of self-perception, non-self-terms that are importantly associated to some other self-terms, processes related to the executive self, and self-views. I also look at how various self-terms may be related, suggesting for instance that self-talk represents a fundamental cognitive activity leading to two possible forms of self-focus, self-reflection and self-rumination, themselves linked in complex ways to a host of other positive (e.g., ToM) or dysfunctional (e.g., self-destruction) self-processes. I view my proposed model both as a look-up table and as a guide to the many ways in which one might experimentally define and operationalize self-variables.

Several self-terms still need to be defined—in particular, self-biases (e.g., self-deception, self-verification), reactions to the self (e.g., self-regard, self-blame), and terms related to the self’s interpersonal style (selfish, self-righteous). One must acknowledge that specific self-related terms may signify slightly different things in various contexts, meaning that in the end it may be difficult, if not impossible, to come to a complete definitional consensus. But a tentative definition is more desirable than no definition at all.

## Author Contributions

The author confirms being the sole contributor of this work and approved it for publication.

## Conflict of Interest Statement

The author declares that the research was conducted in the absence of any commercial or financial relationships that could be construed as a potential conflict of interest.
